# Chronic *In Vivo* Imaging of Ponto-Cerebellar Mossy Fibers Reveals Morphological Stability during Whisker Sensory Manipulation in the Adult Rat^1,2,3^

**DOI:** 10.1523/ENEURO.0075-15.2015

**Published:** 2015-10-22

**Authors:** Daria Rylkova, Aidan R. Crank, David J. Linden

**Affiliations:** The Solomon H. Snyder Department of Neuroscience, Johns Hopkins University School of Medicine, Baltimore, Maryland 21205

**Keywords:** cerebellum, in vivo imaging, mossy fiber

## Abstract

The cerebellum receives extensive disynaptic input from the neocortex via the basal pontine nuclei, the neurons of which send mossy fiber (MF) axons to the granule cell layer of the contralateral cerebellar hemisphere. Although this cortico-cerebellar circuit has been implicated in tasks such as sensory discrimination and motor learning, little is known about the potential role of MF morphological plasticity in the function of the cerebellar granule cell layer. To address this issue, we labeled MFs with EGFP via viral infection of the basal pons in adult rats and performed *in vivo* two-photon imaging of MFs in Crus I/II of the cerebellar hemisphere over a period of several weeks. Following the acquisition of baseline images, animals were housed in control, enriched, or deprived sensory environments. Morphological dynamics were assessed by tracing MF axons and their terminals, and by tracking the stability of filopodia arising from MF terminal rosettes. MF axons and terminals were found to be remarkably stable. Parameters derived neither from measurements of axonal arbor geometry nor from the morphology of individual rosettes and their filopodial extensions significantly changed under control conditions over 4 weeks of imaging. Increasing whisker stimulation by manipulating the sensory environment or decreasing such stimulation by whisker trimming also failed to alter MF structure. Our studies indicate that pontine MF axons projecting to Crus I/II in adult rats do not undergo significant structural rearrangements over the course of weeks, and that this stability is not altered by the sustained manipulation of whisker sensorimotor experience.

## Significance Statement

Two-photon microscopy has allowed the visualization of neuronal morphology and activity in behaving animals, thus making it possible to explore the relationship between structure and experience in the intact nervous system. Ours is the first study to investigate this relationship in the cortico-cerebellar circuit, which is critical for the processing of sensorimotor information. By imaging the arbor of ponto-cerebellar axons, called mossy fibers, in adult rats exposed to sensory-enriched or sensory-deprived environments over weeks, we show that these inputs remain morphologically stable despite alterations in sensory inputs.

## Introduction

While axons in the developing brain show dramatic growth and activity-driven structural plasticity (Witte et al., 1996; Ruthazer et al., 2003; Nishiyama and Linden, 2004; Huberman et al., 2008; Erzurumlu and Gaspar, 2012), the degree to which these processes persist in the adult brain is poorly understood. Recently, two-photon imaging has enabled the monitoring of individual axons over time *in vivo* and has thus permitted the study of structural axonal dynamics under basal conditions as well as in response to perturbations. In the mouse, the axonal arbors and terminals of layer 5 pyramidal neurons across somatosensory, auditory, and visual cortices are largely stable in the absence of sensory manipulation (Majewska et al., 2006). On the other hand, the axons of layer 6 pyramidal cells, branching in layer 1 of barrel cortex, have highly dynamic boutons, with less than half remaining stable over the course of a month (De Paola et al., 2006). Axonal dynamics appear to be cell type specific since boutons on thalamocortical and layer 2/3 pyramidal cell axons innervating the same area of barrel cortex are highly stable over 1 month (De Paola et al., 2006). In the mouse cerebellar cortex, the dynamics of olivo-cerebellar climbing fiber axons are branch specific. Under basal conditions, axonal structure and bouton stability are much lower on transverse branches compared with ascending branches. While transverse branches fail to form conventional asymmetric synapses, ascending branches form conventional synapses with Purkinje cells as well as spillover synapses with interneurons (Nishiyama et al., 2007).

A number of *in vivo* imaging studies have also examined the relationship between the basal dynamics of axonal morphology and dynamics in response to injury or sensory manipulation. In primate visual cortex, binocular retinal lesions result in a rapid (within hours) and sustained (over months) increase in the sprouting of horizontal excitatory axon collaterals into the lesioned zone, which is followed by pruning and an enhanced bouton turnover rate (Yamahachi et al., 2009). On the other hand, the axonal arbors of inhibitory neurons within the lesioned zone sprout outside its borders, presumably to balance increased excitation (Marik et al., 2014). In mouse barrel cortex, sensory deprivation achieved by whisker plucking, results in the sprouting of layer 2/3 pyramidal cell axon collaterals from spared rows into adjacent deprived rows. At the same time, the axonal arbor of inhibitory neurons located in deprived rows dramatically expands into neighboring spared rows (Marik et al., 2010). Both of these changes occur on a timescale similar to that for changes occurring in receptive field properties reported in the cortex (Heinen and Skavenski, 1991; Gilbert and Wiesel, 1992; Marik et al., 2010). While it is well established that axonal structural dynamics contribute to functional changes in the adult cerebral cortex following sensory perturbation, the extent to which circuit-level rearrangements occur in subcortical structures, which also play important roles in sensorimotor processing, is less clear.

**Figure 1.**
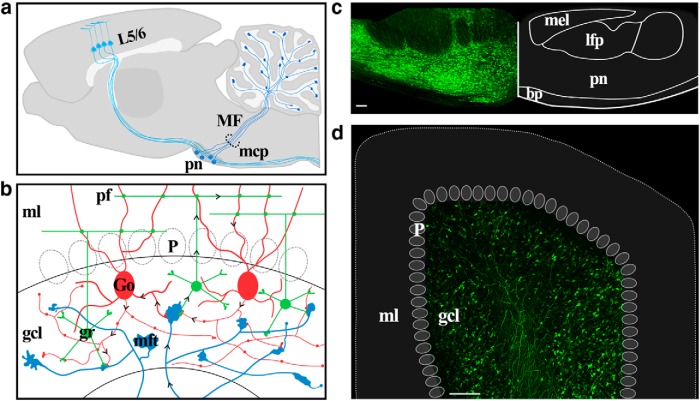
Injection of AAV2/1-EGFP into the basal pons labels ponto-cerebellar mossy fibers in the adult rat. ***a***, A diagram of the cortico-cerebellar circuit. Cortical layer 5/6 pyramidal cells provide ipsilateral input to neurons of the basal pons. Pontine axons travel in the contralateral middle cerebellar peduncle (mcp) and branch throughout the white matter and granule cell layer of the cerebellar hemisphere. ***b***, The granule cell layer microcircuit. Each mossy fiber axon branches over multiple lobules and gives rise to 100–200 terminals. A mossy fiber terminal forms a glomerular synapse with the dendrites of 30–40 granule cells. Granule cells are also innervated by inhibitory Golgi cells. ***c***, EGFP expression in the basal pons revealed by native EGFP fluorescence in a fixed coronal brain slice prepared ∼2 months after the virus injection. This image is a montage of several maximal *z*-projections, each encompassing ∼30 µm of depth. Note the broad distribution of infected cells within the basal pons. The right portion of the panel shows the labeled boundaries of the basal pontine nuclei and surrounding fiber tracts corresponding to approximately −8.0 mm AP from bregma. ***d***, Immunohistochemistry using an antibody directed against EGFP reveals pontine mossy fibers in Crus IIa of the cerebellar hemisphere contralateral to the injection site. The rat was killed, and slices were prepared ∼2 months after viral injection. bp, Brachium pontis; Go, Golgi cell; gcl, granule cell layer; gr, granule cell; mel, medial lemniscus; mft, mossy fiber terminal; ml, molecular layer; lfp, lateral fasciculus of the pons; P, Purkinje cell; pf, parallel fiber; pn, pontine nuclei. Scale bar, 100 μm.

Here we set out to investigate the morphological dynamics of ponto-cerebellar axons, which form the input stage of the cortico-cerebellar circuit. The cerebellum receives a large disynaptic projection from the cerebral cortex via the basal pontine nuclei (Brodal, 1972), which send mossy fiber (MF) axons to innervate the granule cell layer of the cerebellar cortex (Fig. 1*a*). Each MF axon branches to innervate multiple lobules (Mihailoff, 1983) and may, in addition, ramify extensively within each lobule (Palay and Chan-Palay, 1974). While most axons in the brain elaborate small (1-3 µm) boutons, cerebellar MFs have large (5-20 µm) boutons (Palay and Chan-Palay, 1974; Xu-Friedman and Regehr, 2003), called rosettes because of their intricate appearance, that are characterized by petal-like processes emanating from the terminal.

The contribution of somatosensory cortico-cerebellar projections has been demonstrated in the sensory guidance of movement (Jenkinson and Glickstein, 2000), sensory discrimination (Gao et al., 1996), and motor-learning tasks (Galvez et al., 2007). While some studies have examined the functional properties and plasticity of MF–granule cell synapses with regard to sensory stimuli (Garwicz et al., 1998; Rancz et al., 2007; Roggeri et al., 2008), the morphological dynamics of these axons have not been characterized *in vivo*. Here we have sought to measure the structural dynamics of pontine MFs innervating lobules Crus I/II of the cerebellar hemisphere, which have previously been shown to receive pontine inputs innervated by barrel cortex in rats (Bower et al., 1981; Leergaard et al., 2006). We used *in vivo* two-photon imaging in adult rats and tracked the dynamics of MF axons under basal conditions as well as in response to perturbations of the whisker sensory experience of the animal.

## Materials and Methods

### Animals

All animal procedures were approved by the Animal Care and Use Committee of The Johns Hopkins University School of Medicine. Experiments were performed on male Long–Evans rats (Charles River) between postnatal day 25 (P25) and P100. Animals were housed two per cage in a temperature- and humidity-controlled vivarium, and were maintained on a 12 h light/dark cycle (lights off at 6:00 P.M.) until undergoing cranial window surgery, following which they were housed individually. Food and water were freely available throughout the experiment.

### Surgical procedures

Surgeries were performed under isoflurane anesthesia (1-3% in oxygen). Temperature was maintained at ∼37°C with a thermostat-controlled warming blanket (Stoelting). Viral injections and cranial window surgeries were performed at least 1 week apart. ACSF, pH 7.2, which was used during cranial window surgery, contained the following (in mm): 135 NaCl, 5.4 KCl, 1 MgCl_2_, 1.8 CaCl_2_, 5 HEPES.

At P25–P30, rats received unilateral injections of 0.3 µl adeno-associated virus (AAV)2/1-hsynapsin-EGFP-WPRE (University of Pennsylvania Gene Therapy Program Vector Core) in the basal pons (Fig. 1*c*,*d*). Animals were placed into the stereotaxic frame, and given a 0.1 ml, s.c., injection of 2% lidocaine and 1:100,000 epinephrine at the incision site. A small incision, centered on lambda, was made, and the periosteum was cleared. A dental drill was used to make a hole at +0.9 to 1.2 mm anteroposterior (AP) and +2.7 mm mediolateral from lambda. The dorsoventral coordinate (−9.0 mm) was measured from the dura. Injection coordinates were chosen based on anatomical studies of cortico-pontine projections from face sensory cortical regions (Leergaard et al., 2000b; Leergaard et al., 2004). The injection was made at an 11^°^ angle tilted to the left from the vertical axis using a Hamilton syringe (model 7001) over a 5 min period. The syringe was left in place for 10 min after the end of the injection. Kwik-cast silicone (WPI) was used to seal the hole after the syringe was retracted. The skin was then sutured, and the animals were given subcutaneous injections of Baytril (5 mg/kg) and buprenorphine (0.03 mg/kg) to manage infection and pain, respectively.

**Figure 2.**
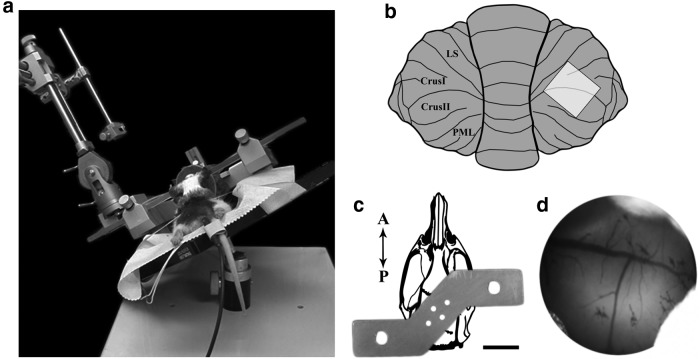
Surgical methods. ***a***, Cranial window surgeries were performed on a modified tilting stereotaxic device to allow for easier access to the lateral cerebellar hemispheres. ***b***, Diagram of the cranial window position overlying lobules Crus I and II. ***c***, Custom headplate used to secure the animal to the imaging apparatus. The headplate is shown overlayed on a drawing of the adult rat skull. Four skull screws and dental cement were used to attach the headplate to the skull. The two holes at the edges of the headplate were used to secure the animal to the imaging stage. Scale bar, 1 cm. ***d***, The surface of the cerebellum viewed through the cranial window. This image is oriented in the same manner as in ***b***.

Approximately 1 week later, animals were prepared with open skull cranial windows overlying the posterior cerebellar hemisphere (Fig. 2), including Crus Ib and IIa, contralateral to the pontine injection site. A Kopf instruments stereotaxic frame was modified to allow tilting, thereby providing easier access to the cerebellar hemispheres. Preoperatively, animals were given subcutaneous injections of dexamethasone (2 mg/kg) and carprofen (5 mg/kg) in order to reduce inflammation. A 1:1 mixture of lidocaine (2 mg/ml) and bupivacaine (2 mg/ml) in epinephrine (1:100,000) was injected subcutaneously at the midline of the head and the caudal perimeter at the level of the ears. A large incision was made, and an oval of skin was removed to expose the muscle overlying the cerebellum. After removing the periosteum and the muscle over the cerebellum and lateral edges of the skull, the skull was cleaned with 3% H_2_O_2_. Surgifoam (Johnson & Johnson) soaked in ACSF was used to stop bleeding from the surrounding tissue. Prior to making the cranial window, two skull screws were placed anterior to the coronal and lambdoid sutures. A custom s-shaped steel head plate (Fig. 2*c*) was affixed to the skull using several layers of Metabond dental cement (Parkell), being careful to avoid the area of the craniotomy. A square craniotomy, slightly larger than the coverslip, was made using a combination of the dental drill (0.5 mm burr) and a scalpel blade (#11). The area was repeatedly rinsed with ACSF to prevent overheating. Once the skull flap was removed, a precut coverslip (2 × 2 mm) was placed directly over the dura and sealed with Metabond dental cement. Baytril (5 mg/kg) and buprenorphine (0.03 mg/kg) were given postoperatively. Baytril (5 mg/kg) and dexamethasone (1 mg/kg) were administered daily for the following 3 d. Animals were allowed to recover for at least 2 weeks before imaging experiments began.

### *In vivo* two-photon imaging

Animals were imaged under isoflurane anesthesia (1–2%). A Zeiss LSM510 NLO equipped with non-descanned detectors was used for two-photon imaging with a Coherent Chameleon Ti:sapphire laser tuned to 920 nm as the excitation source. The imaging stage was tilted such that the cranial window was parallel to the objective. Images were acquired with a 40×, 1.0 numerical aperture water-immersion objective (W Plan-Apochromat, Zeiss). Square image frames were 110–200 µm wide and acquired at a resolution of 0.11–0.44 µm/pixel and a 2 µm *z*-step. Mossy fibers could be resolved from 150–400 µm below the pial surface. Imaging sessions typically lasted an hour. Animals were imaged for up to 2 months at intervals ranging from 1 to 5 d.

### Sensory manipulation

**Figure 3.**
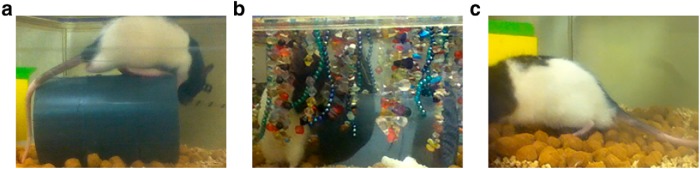
Home-cage conditions for sensory manipulation. ***a***, For control and baseline conditions, animals were given a PVC tube, which they were free to explore. ***b***, The enriched whisker stimulation environment included the PVC tube and a variety of hanging beads. ***c***, For the deprived condition, animals had their whiskers plucked for the duration of imaging, and the PVC tube was removed.

At least two baseline time points were acquired prior to assigning animals to the control (*N* = 3), enriched (*N* = 2), or deprived (*N* = 3) sensory conditions. Under baseline and control conditions, animals had access to a polyvinyl chloride (PVC) tube (2.5 inches in diameter, 6 inches in length), which they were free to explore (Fig. 3*a*). To enhance whisker sensory experience, animals were placed in a cage, which contained the PVC tube as well as strings of hanging beads of various shapes and materials (Fig. 3*b*). To promote exploration, peanut butter was regularly placed on the strings. To deprive animals of whisker sensory experience, their whiskers were plucked bilaterally throughout the experiment and the PVC tube was removed (Fig. 3*c*).

### Fixation and recovery of imaged area

At the end of an imaging experiment, a 5× magnification image of the surface blood vessels was acquired and the locations of the fields of view imaged *in vivo* were noted. Animals were then transcardially perfused with PBS, followed by 4% paraformaldehyde in PBS, and decapitated. The head of the animal was then placed in a stereotaxic frame with the cement head cap and plate in place. The cranial window was removed by drilling around the coverslip. Once the coverslip was removed, a 30 gauge needle dipped in dextran-conjugated Alexa Fluor 555 was used to penetrate the brain at the edges of the craniotomy to create fiduciary marks. The brain was then removed and incubated in 30% sucrose for ∼24 h. The pons was carefully separated from the cerebellum, and 100 µm slices were cut on a freezing microtome. The pons was sectioned in the coronal plane, while the cerebellar hemisphere was sectioned parallel to the cranial window plane. Slices were either directly mounted and imaged or were first immunostained. For immunostaining, slices were washed in Tris-buffered saline [TBS; 3× 5 min at room temperature (RT)], then permeabilized in TBS containing 0.5% Triton X-100 (Sigma; 3× 20 min at RT). Slices were then blocked overnight at 4°C in TBS containing 10% goat serum, 0.5% Triton X-100, and 0.02% sodium azide (Sigma). Chicken anti-GFP primary antibody (Aves Labs) was diluted 1:2000 in the blocking solution, and slices were incubated for 48 h at 4°C. Slices were then washed in blocking solution (5× 30 min) and incubated in secondary antibody (goat anti-chicken conjugated to Alexa Fluor 488 (Jackson ImmunoResearch) at a dilution of 1:500 in blocking solution for an additional 48 h at 4°C. Finally, slices were washed in TBS containing 0.5% Triton X-100 (5× 10 min at RT), followed by TBS alone (3× 10 min at RT), and mounted. Parameters for brain slice imaging were similar to those for *in vivo* imaging, except that an argon laser tuned to 488 nm was used as the excitation source, and images were acquired in single-photon confocal mode.

### Analysis

Images were deconvolved using the blind deconvolution algorithm with adaptive point spread function in Autoquant X version 2.1 (Media Cybernetics). Time points were manually registered using Fiji (ImageJ) software. Deconvolved images were then imported into Imaris version 7.0 software (Bitplane) and analyzed in 3-D-rendered mode. Axons were manually traced using the filament function while the location of terminals was marked using the spots function. A summary of the number of animals and axons we analyzed is presented in Table 1. Custom scripts written in Matlab (MathWorks) were used to import traced axons from Imaris as directed graphs. Functions modified from the Trees toolbox (Cuntz et al., 2011) were then used to quantify axonal geometry. Specifically, we analyzed the change in axon length, number of branch points, length of individual branches, path length between terminals, tortuosity, and convex hull volume. To calculate distances between MF terminals, the *x*, *y*, and *z* coordinates of spots marking their locations were exported from Imaris and assigned the closest node on the graph of the corresponding parent axon. The tortuosity of axonal branches was calculated as the ratio of the Euclidian distance between two end points and the axonal path length between those points. Volume was calculated using the convhulln function in Matlab. To quantify the stability of filopodia emanating from MF rosettes, we selected a random subset of filopodia in multiple volumes and for multiple time points. We then scored the presence of those filopodia in earlier and later time points.

**Table 1.** An overview of the dataset

	Number of animals	Brain volume traced (µm^3^)	Total axon length (µm)	Number of axons	Number of terminals	Number of filopodia
Control	3	1.68 × 10^7^	9,736.42	48	131	129
Enriched	2	8.31 × 10^6^	10,321.37	81	181	154
Deprived	3	9.25 × 10^6^	13,822.74	87	197	138

The number of animals, brain volume, total axonal length, number of individual axons, terminals, and filopodia analyzed for the control and experimental groups.

### Statistics

Imaging days were centered such that the time point immediately preceding sensory manipulation was taken as day 0. For the control group, day 0 was chosen as the midpoint in the imaging timecourse. Data were fit using a generalized linear mixed-effects model (Verbeke and Molenberghs, 2009) of the following form: response ∼ baseline + time * group + (time|axon) + (time|animal).

The baseline measurement, time, experimental group, and the interaction between time and group were included as the fixed effects. A slope and an intercept for each axon (or axonal branch) and animal were included as the random effects. Data distributions, which are indicated in Table 2, were determined by visually comparing the fits of several possible distributions using the dfittool function in Matlab. To test the significance of the fixed effects, a likelihood ratio test was performed to compare a reduced null model with the full model, which included one of the main effects or interaction terms. Functions provided in the Matlab statistics toolbox (glme, compare) were used to fit the data and perform tests, as follows: reduced model: response ∼ baseline + (time|axon) + (time|animal)

**Table 2.** Statistical analysis

	Data structure	Type of test	Power*
a	Inverse Gaussian	GLLM, LR	a
b	Poisson	GLMM, LR	a
c	Gamma	GLMM, LR	a
d	Gamma	GLMM, LR	a
e	Log normal	GLMM, LR	a
f	Log normal	GLMM, LR	a
g	Inverse Gaussian	GLMM, LR	a
h	Poisson	GLMM, LR	a
i	Gamma	GLMM, LR	a
j	Gamma	GLMM, LR	a
K	Log normal	GLMM, LR	a
L	Log normal	GLMM, LR	a
M	Inverse Gaussian	GLMM, LR	a
n	Poisson	GLMM, LR	a
o	Gamma	GLMM, LR	a
p	Gamma	GLMM, LR	a
q	Log normal	GLMM, LR	a
r	Log normal	GLMM, LR	a

Letters in the first column refer to values in the Results section. GLMM, Generalized linear mixed model; LR, likelihood ratio test.

*It is not readily possible to calculate the observed power for generalized linear mixed models with multiple random effects.

For the purposes of displaying the data concerning axonal morphology (see Fig. 7), values were normalized to day 0 and are plotted as the mean ± SEM of the percentage of day 0. In addition, time intervals were grouped into 6-d-long bins.

## Results

We have sought to measure the structure of ponto-cerebellar MFs. Pontine MFs are primarily glutamatergic (Beitz et al., 1986; Ottersen et al., 1990) myelinated axons, which range in diameter from 0.4 to 1.5 µm. Once the axon reaches the granule cell layer, the myelin is interrupted every 20–80 µm at the location of the rosettes (Palay and Chan-Palay, 1974). The MF terminal forms the core of the cerebellar glomerulus (Fig. 1*b*), which also includes the dendrites of granule cells and the dendrites as well as axons of Golgi cells (Gray, 1961; Hámori and Szentágothai, 1966; Palay and Chan-Palay, 1974). The dendritic processes of 20–40 granule cells envelop each rosette (Palkovits et al., 1972; Palay and Chan-Palay, 1974), which may contain up to hundreds of release sites (Xu-Friedman and Regehr, 2003).

**Figure 4.**
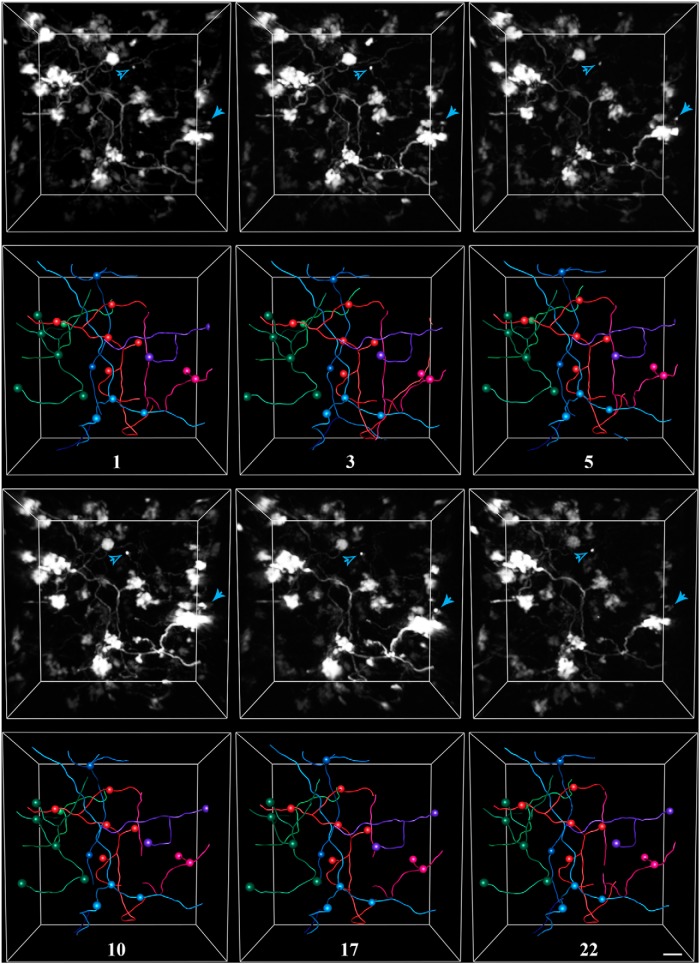
Exemplar *in vivo* images and tracings from a rat housed in control conditions. Pontine mossy fibers expressing EGFP were imaged repeatedly over a 3 week period in the Crus I/II lobules of the cerebellar hemisphere. For each time point, the 3-D-rendered fluorescence image (this is not a *z*-stack projection) is shown in the top panel, and the corresponding tracing is shown in the bottom panel. Spheres mark the locations of axon terminals. Each color represents a separate axon identified within the volume. The number of days since the volume was first acquired is indicated under each set of tracings. Arrowheads indicate examples of two filopodial processes extending from nearby rosettes, which were stable for the duration of imaging. Scale bar, 10 µm.

To examine the morphological dynamics of pontine MFs *in vivo*, we labeled MF axons with EGFP mediated by AAV infection of the basal pons (Fig. 1*c*,*d*). We then repeatedly imaged the cerebellar hemisphere of adult rats through a chronically implanted cranial window over Crus I/II (Fig. 2*b*,*d*). Pontine MFs enter the cerebellum via the medial cerebellar peduncle (Fig. 1*a*) and branch into multiple lobules to give rise to tens of intricate rosettes within each lobule (Palay and Chan-Palay, 1974; Mihailoff, 1983). We investigated both the large-scale dynamics of the axonal arbor as well as the fine-scale morphology of the MF rosette over timescales ranging from minutes to weeks. Figure 4 shows an example volume of MFs imaged over the course of 3 weeks in an animal housed in standard conditions, which are pictured in Figure 3*a*. Movie 1 shows an animation of a single time point of this volume. Examining the volume by visual inspection indicated that the same MF rosettes were present for all time points and that the large-scale architecture of the axonal arbor did not change. To assess the stability of the arbor, we quantified the following six variables relating to axonal structure over time: total axonal length, branch length, number of branch points, interterminal axonal path length, axonal tortuosity, and convex hull volume. All six of these metrics remained stable over time (see Fig. 7).

The finer structure of the MF terminal was characterized by tracking the presence of individual filopodial processes extending from rosettes. Large MF rosettes often have both short (2–10 µm) protrusions as well as longer (>20 µm) processes, whose endings also contain release sites (Palay and Chan-Palay, 1974). Two of these filopodia are indicated by arrowheads in Figure 4. Both the filopodia indicated by the open arrowhead, which extends ∼50 µm from its terminal, and that indicated by the filled arrowhead, which extends 10 µm from its terminal, are present for the duration of imaging. Of the 129 identified filopodia in three animals, only 1 was observed to be dynamic over a 3 week period.

Electrophysiological mapping studies of Crus I/II in rats have shown that these regions process perioral and vibrissal sensorimotor information (Shambes et al., 1978; Shumway et al., 1999, 2005; Proville et al., 2014), and are active when the animal is exploring its environment (Hartmann and Bower, 2001). Although the size of cerebellar patches concerned with different perioral regions varies between animals, the medial aspect of Crus II is consistently responsive to whisker stimulation (Bower and Kassel, 1990). Moreover, cortico-ponto-cerebellar inputs have been shown to contribute to sensory responses recorded in Crus I/II and project to the same regions as trigemino-cerebellar inputs (Bower et al., 1981; Morissette and Bower, 1996). To further probe the stability of pontine mossy fibers and explore the role of the cortico-cerebellar system in sensory processing and integration, we tested the impact of a global sensory manipulation on morphological dynamics, by housing animals in conditions where whisker stimulation would either be enhanced or diminished (Fig. 3). To increase whisker and facial stimulation in the enriched condition, animals were placed into a cage filled with many hanging beads of various sizes and textures (Fig. 3*b*). On the other hand, animals in the deprived condition (Fig. 3*c*) had their whiskers plucked after the acquisition of baseline images, and were continuously plucked for several weeks of imaging.

**Figure 5.**
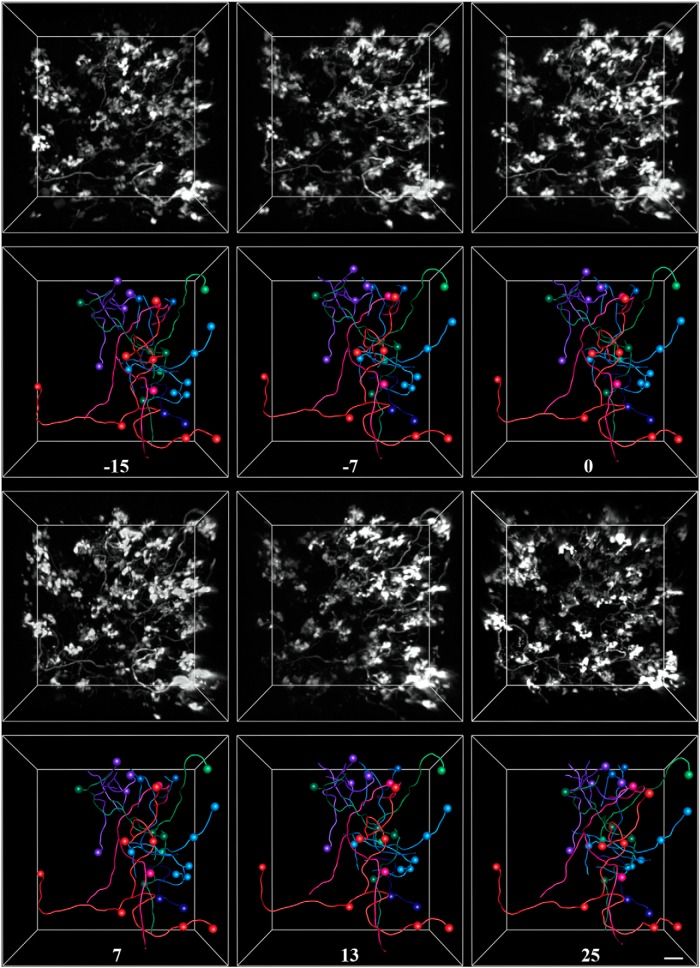
Exemplar *in vivo* images and tracings from a rat housed in enriched conditions. Pontine mossy fibers expressing EGFP were imaged repeatedly over a 6 week period in the Crus I/II lobule of the cerebellar hemisphere. For each time point, the 3-D-rendered fluorescence image is shown in the top panel, and the corresponding tracing is shown in the bottom panel. Spheres mark the locations of axon terminals. Each color represents a separate axon identified within the volume. The number of days since the animal was introduced to the enriched environment is indicated under each set of tracings. Scale bar, 10 µm.

**Figure 6.**
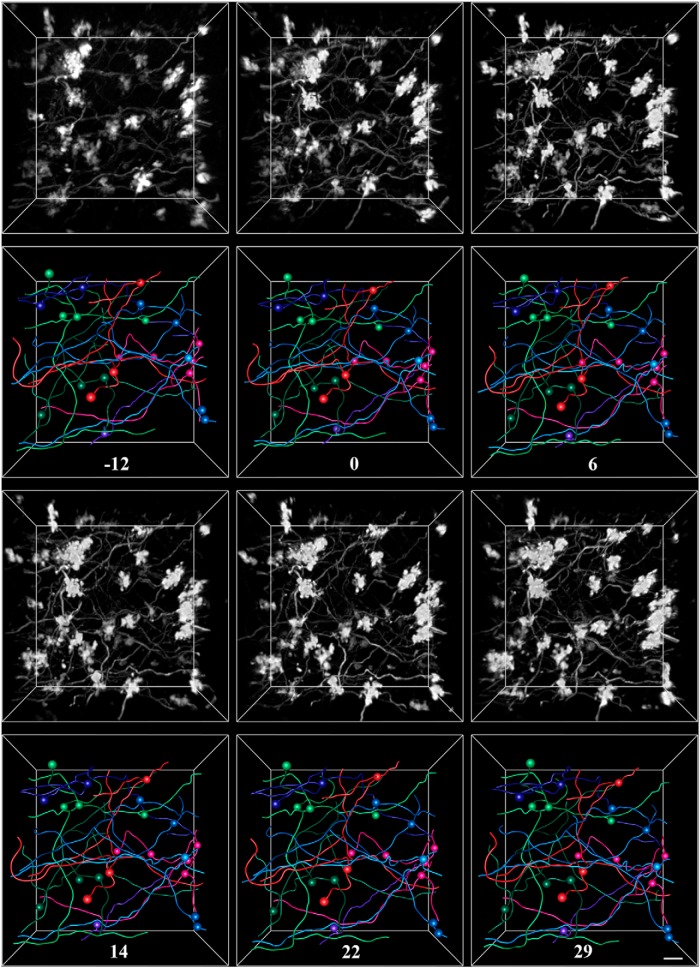
Exemplar *in vivo* images and tracings from a rat housed in deprived conditions. Pontine mossy fibers expressing EGFP were imaged repeatedly over a 6 week period in the Crus I/II lobule of the cerebellar hemisphere. For each time point, the 3-D-rendered fluorescence image is shown in the top panel, and the corresponding tracing is shown in the bottom panel. Spheres mark the locations of axon terminals. Each color represents a separate axon identified within the volume. The number of days since the animal was introduced to the deprived environment and had whiskers plucked, is indicated under each set of tracings. Scale bar, 10 µm.

**Figure 7.**
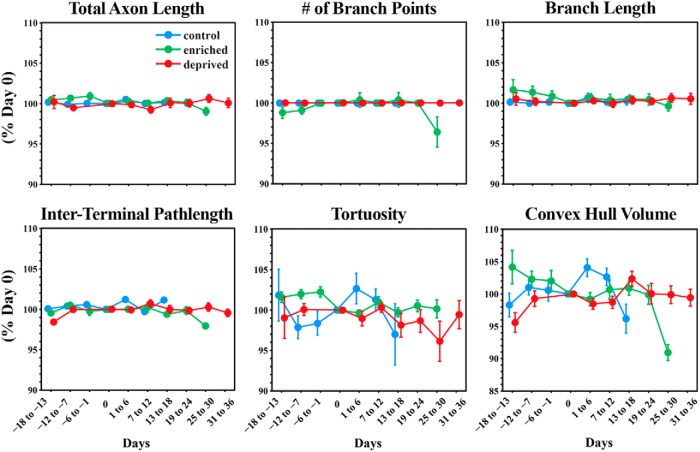
Ponto-cerebellar mossy fiber axons are stable in control, enriched, and deprived conditions. Mossy fiber axons were manually traced for each imaging time point. Tracings were used to quantify the morphological parameters of individual mossy fiber axons across time. For the purposes of displaying data, time points were aligned by collecting data into 6 d time bins. Day 0 is the day animals were introduced into sensory enriched or deprived housing conditions. For the control group, day 0 was taken as the approximate midpoint of imaging. Data were normalized and are reported as the percentage of day 0. Data are presented as the mean ± SEM. Total axon length shows the mean percentage change in the total length of individual axons. The number of branch points refers to the total number of branch points per axon. Branch length was measured for individual axonal branches. Interterminal path length refers to the axonal path length separating two terminals. Tortuosity was calculated as the ratio of the Euclidian distance between two terminal points and the axonal path length between those points of individual axons. The term “convex hull volume” refers to the convex hull volume of individual axons.

MF axons were imaged repeatedly and traced for animals in both the enriched and deprived conditions. Figures 5 and 6 show exemplar volumes of MFs imaged in an enriched and deprived animal, respectively. The manipulation, which is indicated as day 0 in both examples, began ∼2 weeks after the start of imaging, and continued for at least 3 weeks, depending on the cranial windows remaining clear. All MF rosettes present at the start of imaging were still present at the last time point. As with control animals, we quantified the stability of MF axons in sensory-manipulated groups by measuring axonal length, the number of branch points, the length of individual branches, interterminal path length, tortuosity, and the volume of each axon over time (Fig. 7). We then fit the data using a mixed-effects model, and examined the effect of time, group, and interaction between time and group on each variable. We defined day 0 as the day when the sensory environment of animals was altered or, in the case of control animals, a day approximately half-way through the imaging period. Similarly to animals in control conditions, pontine MF axons of animals housed in enriched or deprived environments remained remarkably stable before and for weeks after the change in environment. We found that there was no effect of time on the total length of each axon [χ^2^(1) = 2.04, *p* = 0.16]^a^, the number of branch points per axon [χ^2^(1) = 0.77, *p* = 0.31]^b^, the length of individual branches [χ^2^(1) = 0.77, *p* = 0.38]^c^, path-length distance between MF terminals [χ^2^(1) = 0.30, *p* = 0.58]^d^, tortuosity [χ^2^(1) = 0.005, *p* = 0.94]^e^, and volume [χ^2^(1) = 1.30, *p* = 0.20]^f^. There was no effect of treatment on length [χ^2^(2) = 1.78, *p* = 0.36]^g^, the number of branch points [χ^2^(2) = 1.93, *p* = 0.36]^h^, length of branches [χ^2^(2) = 1.93, *p* = 0.38]^i^, the distance between terminals [χ^2^(2) = 2.53, *p* = 0.28]^j^, tortuosity [χ^2^(2) = 0.42, *p* = 0.81]^k^, or volume [χ^2^(2) = 1.93, *p* = 0.47]^l^. There was no interaction effect on length [χ^2^(2) = 3.05, *p* = 0.12]^m^, the number of branch points [χ^2^(2) = 3.84, *p* = 0.07]^n^, the length of branches [χ^2^(2) = 3.84, *p* = 0.15]^o^, the distance between terminals [χ^2^(2) = 4.58, *p* = 0.10]^p^, tortuosity [χ^2^(2) = 0.30, *p* = 0.86]^q^, and volume [χ^2^(2) = 2.10, *p* = 0.29]^r^.

**Figure 8.**
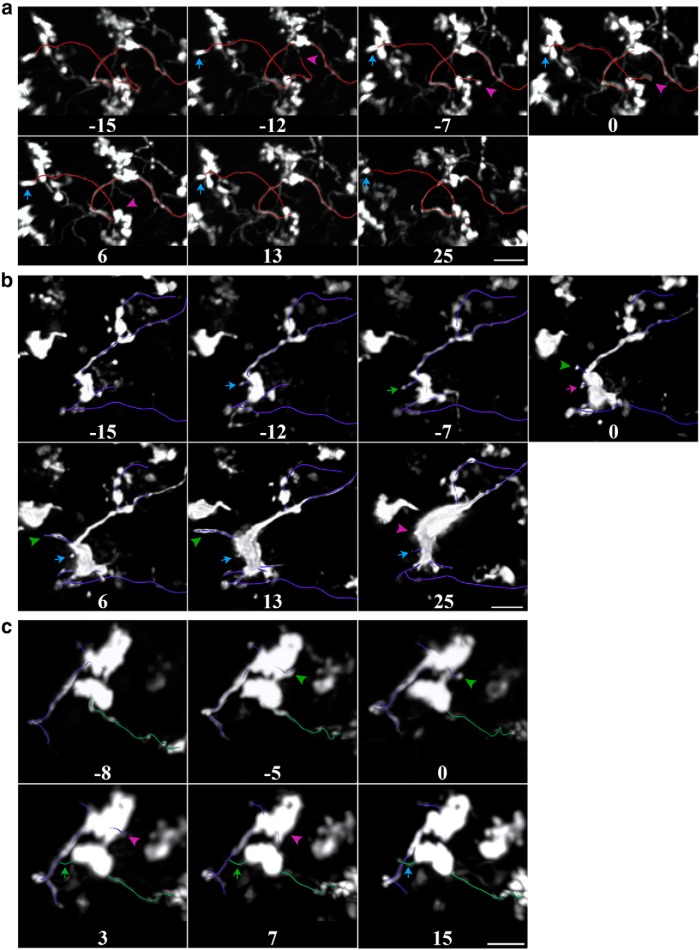
Examples of rare mossy fiber branch and filopodial dynamics captured *in vivo*. ***a***, Time series showing the retraction of an axonal branch over a 2–3 week period. The large mossy fiber terminal at the center gives rise to two branches ending in small terminals. The branch to the left (arrow) remains stable, whereas the one on the right (arrowhead) retracts. ***b***, ***c***, Examples of two dynamic filopodia (arrow and arrowhead) on a single mossy fiber terminal. Blue, stable; magenta, retracting; green, extending compared with the previous time point. All examples are snapshots of the 3-D-rendered volume and were taken from the enriched group. Days since the animal was introduced to the enriched environment are indicated at the bottom of each image. Scale bar, 10 µm.

**Figure 9.**
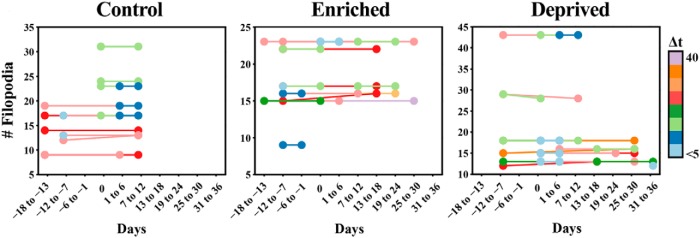
Filopodia emitted from mossy fiber terminals are stable in control, enriched, and deprived conditions. A subset of filopodial protrusions arising from mossy fiber terminal rosettes were marked in several volumes and time points for each animal. The panels show the number of filopodia identified in one time point that were also present in a second time point for the control, enriched, and deprived groups, respectively. The time interval between comparisons is color coded. With few exceptions, all filopodia identified in one time point were also present in another time point regardless of the time interval. The number of added or lost filopodial protrusions never exceeded one in a given imaging volume.

In addition to examining gross axonal structure, we also tracked individual filopodial processes emanating from MF terminals (Figs. 8, 9). These filopodial processes have previously been shown to be dynamic in both hippocampal and cerebellar MFs (Galimberti et al., 2006; Ruediger et al., 2011). Apart from a small fraction of terminals where we could see the appearance (Fig. 8*b*,*c*), morphing (Fig. 8*b*), or disappearance (Fig. 8*a*) of filopodia, the vast majority remained stable. Importantly, rare dynamic filopodia could be observed before day 0 and therefore did not seem to be associated with sensory manipulations (Fig. 9).

## Discussion

The aim of our experiments was to investigate the morphological plasticity of ponto-cerebellar MF axons in rats using *in vivo* two-photon imaging. Since these axons are the major source of neocortical information to the cerebellum, understanding the dynamics of these axons sheds light on properties of cerebro-cerebellar computations. We observed that the axonal arbor architecture and terminal morphology of MF axons projecting to cerebellar lobules Crus I/II of adult rats remain stable over the course of weeks. Furthermore, this stability is not altered when animals are housed in an environment in which whisker sensorimotor experience is either enriched or deprived.

Although the basal pons receives input from diverse sources, including collaterals of other pontine cells (Mihailoff, 1978), the cerebellar nuclei (Lee and Mihailoff, 1990), red nucleus, and trigeminal and spinal cord nuclei among others (Mihailoff et al., 1989), these cells have been suggested to be most strongly driven by activity in the cerebral cortex (Allen and Tsukahara, 1974). In rats, the vibrissal region of the motor cortex provides bilateral input to the basal pontine nuclei, indicating that this corticocerebellar circuit is involved in the modulation of bilateral whisker movements (Alloway et al., 2010). Tracer studies examining the terminal fields of efferents from neighboring sites in barrel cortex, have observed the largest degree of overlap in the basal pons compared with other subcortical targets (Leergaard et al., 2000a; Hoffer et al., 2005). This, along with electrophysiological evidence that individual pontine cells may be driven by convergent cortical inputs both within and across sensory modalities (i.e., somatosensory and auditory; Potter et al., 1978), indicates that the pontine nuclei serve to integrate information before it is sent to the cerebellum, rather than act as a simple conduit. On the other hand, the high density of cortico-pontine terminals (Hoffer et al., 2005) and fast conduction velocity of cortico-pontine and ponto-cerebellar axons (Allen et al., 1975) suggest that the pontine nuclei are also efficient at transforming cortical signals into a form useful for the cerebellum, which is supported by the high coherence between the cerebellum and barrel cortex during whisking (O'Connor et al., 2002).

While numerous studies (Polley et al., 2004; Breton and Stuart, 2009; Oberlaender et al., 2012) have investigated the effect of sensory manipulations on the structure and function of the cerebral cortex, few have focused on downstream targets, including the cerebellum. Although the effect of whisker deprivation on barrel cortex activity is strongly dependent on the pattern and extent of whisker removal or trimming (Wallace and Fox, 1999; Feldman and Brecht, 2005), deprivation of a sensory input in adulthood generally results in the depression of responses to deprived inputs, and the expansion or strengthening of neighboring, nondeprived inputs over the course of days to weeks (Drew and Feldman, 2009; Marik et al., 2010; Margolis et al., 2012). On the other hand, exposing animals to an enriched, naturalistic environment sharpens the receptive field properties of cells in the barrel cortex and results in the contraction of each whisker representation (Polley et al., 2004). Since pontine cells are likely driven by multiple whiskers (Leergaard et al., 2000a) or even multiple body regions (Potter et al., 1978), it is difficult to predict how manipulations of whisker experience would alter the activity impinging on individual pontine neurons.

In studies where the transection of the trigeminal nerve was used to investigate cerebellar map plasticity, recordings in the granule cell layer revealed changes in map organization, which could only be explained by plasticity in afferent structures, specifically the cerebral cortex, rather than by plasticity intrinsic to the cerebellum (Shumway et al., 1999; Shumway et al., 2005). In those experiments, changes in the receptive fields of the Crus II granule cell layer reflected the statistics of changes in neocortical receptive fields, rather than the somatotopy of Crus II before transection, which was suggested to support the idea that the pattern of MF afferents is hard wired since they enter the cerebellum prior to the appearance of their target granule cells (Bower and Kassel, 1990).

However, there are several lines of evidence supporting the potential for MF axons to undergo structural as well as functional plasticity. Data obtained in slices and *in vivo* have shown that MFs express presynaptic LTP and LTD in response to either electrical or sensory burst stimulation (Maffei et al., 2002; Sola et al., 2004; Roggeri et al., 2008; D'Errico et al., 2009). Animals exposed to cued fear conditioning or rotarod training have greater numbers of filopodial processes per MF terminal in the cerebellar vermis compared with controls (Ruediger et al., 2011). Moreover, animals housed in an enriched environment for 20 d have greater densities of MF terminals and branch points per axon in the cerebellar vermis compared with controls (Vittori, 2009). Discrepancies between this study and ours may be due to several factors, including species (mouse vs rat), the source of MFs (pontine plus spinal plus vestibular vs pontine), cerebellar region (vermis vs hemisphere), and comparison (between vs within animal). While we did not observe the appearance or disappearance of MF terminals, or other structural changes correlated to sensory manipulation, we did observe some rare examples of changes to filopodial processes, suggesting that these axons are capable of undergoing structural rearrangements in adulthood. In the present experiments, we only labelled pontine mossy fibers. It would, however, be important to examine morphological as well as functional dynamics of both pontine and trigeminal/spinal mossy fibers in response to whisker and other sensory manipulations to understand how these sources of sensory information contribute to the sensorimotor processing of the cerebellum.

There are several caveats that should be sounded in interpreting the present findings. First, our experiments focused on adult animals. Thus, it remains possible that ponto-cerebellar MFs show morphological plasticity earlier (or perhaps even later) in life. Second, our analysis was limited to the Crus I/II region, leaving open the possibility that pontine MFs projecting to other regions of the hemispheres or the vermis might behave differently. Third, we did not record neuronal activity along the cortico-cerebellar pathway and did not assess the response properties of those MFs, whose structure we examined over time. For these reasons, it is difficult to speculate the degree to which the sensory manipulations altered firing patterns in the whisker cortico-cerebellar circuit, or the fraction of MFs that provided whisker sensorimotor information. A related issue is that we did not explicitly measure the relevant motor behaviors (whisking, licking, chewing, facial twitches) across the three environmental conditions. While we do not know the extent to which depriving the animal of whiskers and decreasing the number of objects in its cage or substantially increasing the density of objects changed the quantity and quality of movements, it is likely that these conditions differed in both sensory and motor dimensions.

Finally, it is possible that the resolution of *in vivo* imaging did not allow us to detect small changes in the morphology of the terminal, which could impact the number or distribution of release sites. Thus, while we can conclude that MFs projecting from the pons to Crus II are almost entirely stable at the level of light microscopy, we cannot speculate as to functional stability either intrinsic to the MF terminal or extrinsic, arising from plasticity in afferent structures. This is especially true since structural and functional stability are not always correlated. For example, until recently it was believed that sensory cortical plasticity in adult animals following deprivation is primarily restricted to layer 2/3 because the response properties of layer 4 cells are unchanged. However, by combining recordings from layer 4 neurons with the reconstruction of thalamocortical axons, Oberlaender et al. (2012) found that structural changes in thalamocortical axons in layer 4 mask compensatory functional changes following whisker deprivation.

While the experiments of Shumway et al. (1999, 2005) addressed the plasticity of cerebellar somatotopy, they did not examine either structural or functional plasticity on a finer scale. In particular, precerebellar nuclei, which receive inputs from a different body region following deafferentation, may consequently receive different patterns of activity depending on which region ends up “filling in” for the deprived region in the cerebral cortex and how the statistics with which the body part interacts with the external world differ (e.g., high-frequency whisking vs lower-frequency forepaw manipulation). Alterations in cortical circuitry and intrinsic properties of layer 5 corticopontine projection cells may be expressed as changes in spike frequency or timing impinging on the pontine nuclei. Since MFs are capable of undergoing both short-term forms (Saviane and Silver, 2006) and long-term forms (Maffei et al., 2002; D'Angelo et al., 2005; Roggeri et al., 2008) of presynaptic plasticity, further investigation into the functional properties of pontine as well as other sources of MFs (e.g., trigeminal nuclei, nucleus reticularis tegmenti pontis, lateral reticular nucleus, vestibular nuclei) that relay homologous sensory information will be required to further illuminate sensory-driven dynamics in the cerebellum.

Movie 1. 3-D rendering of mossy fibers and traced axons. A single time point from the volume is shown in Figure 4. The animation begins by stepping through each frame of the volume, beginning at ∼150 µm and ending at ∼250 µm below the pial surface. The animation then zooms into multiple terminals. Axonal traces then appear superimposed, followed by the tracings of two filopodial processes on the pink axon, which are also indicated in Figure 4 10.1523/ENEURO.0075-15.2015.video.1
